# The choice between p53-induced senescence and quiescence is determined in part by the mTOR pathway

**DOI:** 10.18632/aging.100160

**Published:** 2010-06-25

**Authors:** Lioubov G. Korotchkina, Olga V. Leontieva, Elena I. Bukreeva, Zoya N. Demidenko, Andrei V. Gudkov, Mikhail V. Blagosklonny

**Affiliations:** Department of Cell Stress Biology, Roswell Park Cancer Institute, BLSC, L3-312, Buffalo, NY 14263, USA

**Keywords:** p53, senescence, rapamycin, mTOR, cancer, cell cycle

## Abstract

Transient induction of p53 can cause reversible quiescence and irreversible senescence. Using nutlin-3a (a small molecule that activates p53 without causing DNA damage), we have previously identified cell lines in which nutlin-3a caused quiescence. Importantly, nutlin-3a caused quiescence by actively suppressing the senescence program (while still causing cell cycle arrest). Noteworthy, in these cells nutlin-3a inhibited the mTOR (mammalian Target of Rapamycin) pathway, which is known to be involved in the senescence program. Here we showed that shRNA-mediated knockdown of TSC2, a negative regulator of mTOR, partially converted quiescence into senescence in these nutlin-arrested cells. In accord, in melanoma cell lines and mouse embryo fibroblasts, which easily undergo senescence in response to p53 activation, nutlin-3a failed to inhibit mTOR.  In these senescence-prone cells, the mTOR inhibitor rapamycin converted nutlin-3a-induced senescence into quiescence. We conclude that status of the mTOR pathway can determine, at least in part, the choice between senescence and quiescence in p53-arrested cells.

## Introduction

Depending on the cell type and other factors p53 activation can result in apoptosis, reversible (quiescence) and irreversible (senescence) cell cycle arrest [1-8]. While the choice between apoptosis and cell cycle arrest has been intensively scrutinized, the choice between quiescence and senescence was not systematically addressed and remains elusive.  In order to observe whether p53 activation causes either senescence or quiescence, others and we employed nutlin-3a. Nutlin-3a, a small molecular therapeutic, inhibits Mdm2/p53 interaction and induces p53 at physiological levels without causing DNA damage [9-11]. It was reported that nutlin-3a caused senescent morphology and permanent loss of proliferative potential [12,13]. However, in other cell lines nutlin-3a caused quiescence so that cells resumed proliferation, when nutlin-3a was removed [14-16].  Moreover,  we recently reported that in human fibroblasts (WI-38tert) and fibrosarcoma cells (HT-1080-p21-9), in which nutlin-3a caused quiescence [16], p53 acted as a suppressor of senescence [17]. Thus, ectopic expression of p21 in these cells caused senescence, while simultaneous induction of p53 converted senescence into quiescence [17]. In agreement with previous reports [18-20], we found that p53 inhibited the mTOR pathway [17]. Importantly, the mTOR pathway is involved in cellular senescence [21-26]. We suggested that p53-mediated arrest remains reversible as long as p53 inhibits mTOR. If this model is correct, then senescence would occur in those cells, in which p53 is incapable of suppressing mTOR. Here we provide experimental evidence supporting this prediction and demonstrate that irreversibility of p53-mediated arrest may result from its failure to suppress the mTOR pathway.

## Results

### Depletion of TSC2 favors senescence by p53

**Figure 1.**
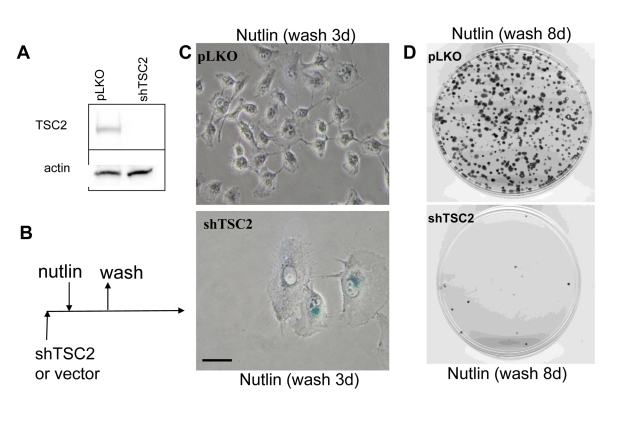
Depletion of TSC2 converts quiescence into senescence in HT-p21-9 cells. (**A**) HT-p21-9 cells were transduced with control lentivirus (pLKO) or lentivirus expressing shTSC2  (sequence # 10) and selected with puromycin for 5 days and then immunoblot was performed. (**B**) Schema: Testing the reversibility of nutlin-3a effects. (**C**) HT-p21-9 cells were transduced with control pLKO or shTSC2 and 5000 cells were plated in 24-well plates and, the next day, were treated with 10 uM nutlin-3a for 3 days. Then nutlin-3a was washed out and the cells were cultivated in fresh medium for 3 days and then stained for beta-Gal and microphotographed. Bars 50 um. (**D**) HT-p21-9 cells were transduced with control pLKO or shTSC2  (and selected for 4 days with puromycin). Then 1000 cells were plated per 60-mm dishes and, the next day, were treated with nutlin-3a for 3 days. Then nutlin-3a was washed out and cells were cultivated in fresh medium for 8 days.  Colonies were stained with crystal violet.

We have shown that nutlin-3a caused quiescence in HT-p21-9 cells and WI-38tert cells [16]. In these cells, nutlin-3a actively suppressed senescence and this suppression was associated with inhibition of the mTOR pathway by p53 [17].  Next, we investigated whether nutlin-3a can cause senescence in cells lacking  tuberous sclerosis 2 (TSC2) (Figure 1A), given that regulation of mTOR by p53 requires TSC2 [18]. The transduced cells were transiently treated with nutlin-3a as shown (Figure 1B). The Tsc2-depleted cells acquired a large/flat morphology and could not resume proliferation, whereas cells treated with vector and nutlin-3a did not become senescent and resumed proliferation, forming colonies after removal of nutlin-3a (Figure 1C-D). The potency of shTSC2 with different sequences varied and two other shTSC2 were less potent but still depleted TSC2 at some time points (Supplemental Figure 1) and partially decreased the proliferative potential in nutlin-3a-arrested cells (Supplemental Figure 1).

**Figure 2.**
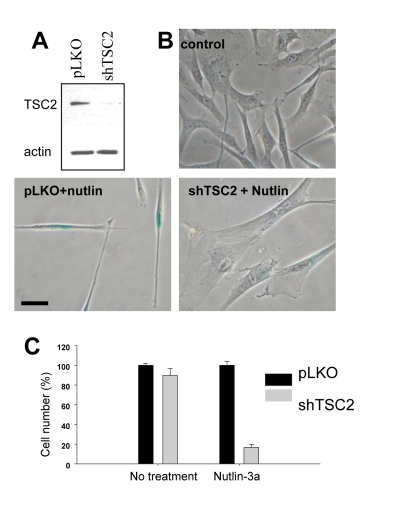
Depletion of TSC2 converts quiescence into senescence in WI-38tert cells. (**A**)Immunoblot. WI-38tert cells were transduced with shTSC or control pLKO and cultured for 5 days. (**B**) WI-38tert cells were transduced with lentiviruses. Next day, medium was replaced and Nutlin (10 uM) with our without rapamycin was added. After 4 days cells were washed and stained for beta-Gal. Bars 50 um. (**C**)  WI-38tert cells were transduced with lentiviruses. Next day, medium was replaced and Nutlin (10 uM) was added. After 4 days cells were washed and counted after 6 days.

We next extended this observation to WI-38tert cells transduced with shTSC2 (Figure 2A). In control, nutlin- 3a caused a lean morphology, a characteristic of quiescence [16]. Depletion of TSC2 by shTSC2 converted quiescent morphology to senescent morphology (Figure 2B).  Furthermore, this was associated with permanent loss of proliferative potential (Figure 2C). In control, cells resumed proliferation after removal of nutlin-3a, whereas nutlin-3a caused permanent loss of proliferative potential in shTSC2-treated cells (Figure 2C). In agreement with our results, it was previously observed that knockout of Tsc2 cooperates with p53 in induction of cellular senescence in MEFs [27].

### Nutlin-3 causes senescence in Mel-10 and -9 cells

**Figure 3.**
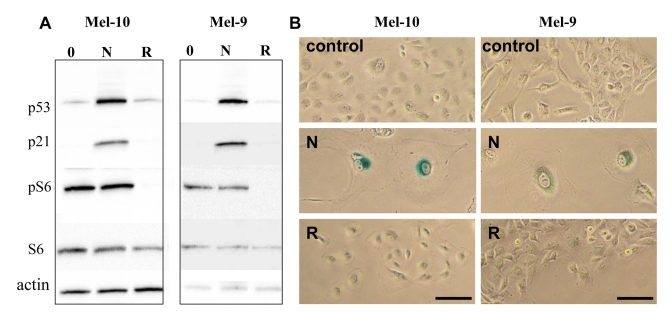
Effects of nutlin-3a and rapamycin on melanoma cells. (**A**) Mel-10 and Mel-9 cells were incubated with 10 uM nutlin (N) and 500 nM rapamycin (R) for 1 day and immunoblot was performed. (**B**) Mel-10 and Mel-9 cells were incubated with 10 uM nutlin and 500 nM rapamycin for 4 days, then drugs were washed out and cells were incubated for additional 4 days and stained for beta-Gal. Bars 50 um.

We next wished to identify senescence-prone cells, which undergo senescence in response to nutlin-3a.  In MEL-10 and Mel-9, two melanoma-derived cell lines, nutlin-3a induced p53 and p21 (Figure 3A) and caused senescent morphology (Figure 3B) and cells did not resume proliferation, when nutlin-3a was removed (Supplemental Figure 2). In contrast, rapamycin did not cause senescent morphology and cells resumed proliferation, when rapamycin was removed (Figure 3B and Supplemental Figure 2). Unlike rapamycin, nutlin-3a did not inhibit S6 phosphorylation (Figure 3A), a marker of rapamycin-sensitive mTOR activity.

**Figure 4.**
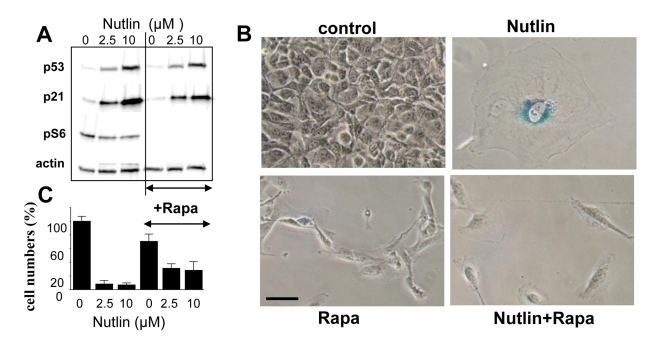
EEffect of rapamycin on nutlin-induced senescence in melanoma cells. (**A**) Mel-10 cells were incubated with 2.5 and 10 uM nutlin with or without 500 nM rapamycin for 1 day and then immunoblot was performed. (**B**) Beta-Gal staining. Mel-10 cells were incubated with 10 M nutlin alone and 500 nM rapamycin for 4 days, then drugs were washed out and cells were incubated for additional 3 days and stained for beta-Gal. Bars 50 um.

### Rapamycin suppresses nutlin-3a-induced senescence

 To establish a causal link between mTOR and senescence, we next investigated whether inhibition of the mTOR pathway by rapamycin could convert nutlin-3a-induced senescence into quiescence. Rapamycin did not affect p53 and p21 induction caused by nutlin-3a but abrogated S6 phosphorylation (Figure 4A), associated with conversion from senescent morphology to quiescent morphology (Figure 4B).  Importantly, cells were capable to resume proliferation following removal of nutlin-3a and rapamycin, indicating that the condition was reversible (Figure 4C). Similar results were obtained with Mel-9 cells (data not shown).

**Figure 5.**
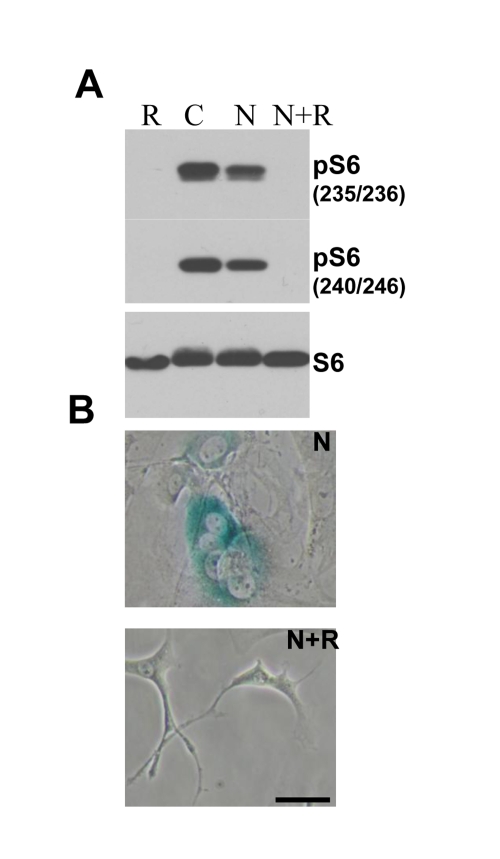
Effect of rapamycin on nutlin-induced senescence in melanoma cells **.  (**A. ) ) Immunoblot. MEF cells were incubated with 10 nutlin-3a with or without 10 nM rapamycin for 1 day and immunoblot using rabbit anti-phospho-S6 (Ser240/244) and (Ser235/236) and mouse anti-S6 was performed. (**B**) Beta-Gal staining. MEF cells were incubated with 10 uM nutlin alone or with 500 nM rapamycin for 4 days, then drugs were washed out and cells were incubated for additional 4 days and stained for beta-Gal. Bars 50 um**.**

Next, we extended this observation to cells of different tissue and species origin. As shown previously, nutlin-3a caused senescence in mouse embryonic fibroblasts (MEFs) [13]. Here we showed that nulin-3a failed to inhibit mTOR pathway in MEF (Figure 5A), and caused senescence (Figure 5B). Rapamycin inhibited the mTOR pathway and converted senescent morphology to quiescent morphology (Figure 5). This suggests that failure to suppress a rapamycin-sensitive pathway determines nutlin-3a-induced senescence instead of quiescence.

## Discussion

**Figure 6.**
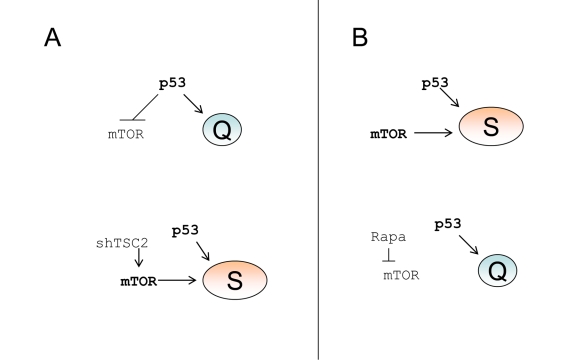
p53 causes senescence by failing to suppress senescence. (**A**) Quiescence-prone cells. Upper panel. P53 causes cell cycle arrest and inhibits the mTOR pathway, thus ensuring quiescence. Lower panel. Transduction of cells with shTSC2 activates mTOR thus converting quiescence into senescence. (**B**) Senescence-prone cells. Upper panel. P53 causes cell cycle arrest without inhibiting the mTOR pathway, thus ensuring senescence. Lower panel. Rapamycin inhibits mTOR thus converting senescence into quiescence.

**Figure 7.**
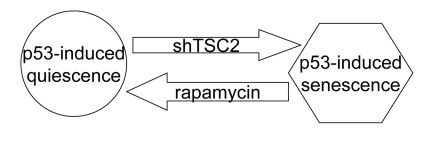
Activation of the mTOR pathway favors senescence in nutlin-3a-arrested cells.

The role of p53 in organismal aging and longevity is complex [28-32], indicating that p53 may act as anti-aging factor in some conditions. We have recently demonstrated that p53 can suppress cellular senescence, converting it into quiescence [17]. In these quiescence-prone cells, p53 inhibited the mTOR pathway, which is involved in senescence program (Figure 6A). Still p53 induces senescence in numerous cell types. Here we showed that in those cell types, in which nutlin-3a caused senescence, it failed to inhibit the mTOR pathway (Figure 6B). The role of active mTOR as a senescence-inducing factor in these cells was demonstrated by using rapamycin, which partially converted nutlin-3a-induced senescence into quiescence (Figure 6B, lower panel). This indicates that rapamycin-sensitive mTOR activity is necessary for senescence during nutlin-3a-induced cell cycle arrest. And vice versa, in quiescence-prone cells, depletion of TSC2 converted quiescence into senescence (Figure 6A, lower panel). Taken together, data suggest that activation of the mTOR pathway favors senescence (Figure 7). In agreement, Ras accelerated senescence in nutlin-arrested cells [13]. Similarly, activation of Ras and MEK in murine fibroblasts converted p53-induced quiescence into senescence [33]. Interestingly, p53 levels did not correlate with the senescence phenotype, suggesting that factors other than p53 may determine senescence [33]. These important observations are in agreement with our model that senescence requires two factors: cell cycle arrest caused by p53 and simultaneous activation of the growth-promoting mTOR pathway (Note: Ras is an activator of the mTOR pathway). And vice versa it was observed that induction of p53 maintains quiescence upon serum starvation, without causing senescence [34]. In agreement, our model predicts that, by deactivating mTOR, serum starvation prevents senescence.

Another factor that favors senescence is the duration of cell cycle arrest [13,35]. Importantly, the duration of the arrest may exceed the duration of treatment with nutlin-3a because of persistent induction of p21 even after removal of nutlin-3a in some cancer cell lines [35]. Additional pathways may be involved in the senescence program. For example, nutlin-3a induces cytoskeletal rearrangement [36]. We speculate that p53 affects not only rapamycin-sensitive mTORC1 but also the mTORC2 complex, given that mTORC2 controls the actin cytoskeleton [37]. Also, p53 inhibits downstream branches of the mTOR pathway [38,39]. P53 stimulates autophagy [18,40], which in turn is essential for life-extension by pharmacological manipulations (see [41-44]). Finally, p53 affects cellular metabolism [45-48] and this effect may contribute to suppression of cellular senescence and synergistically potentate metabolic changes caused by mTOR inhibition. The relative contribution of all these mutually dependent factors needs further investigations. The key role of mTOR in cellular senescence links cellular and organismal aging and age-related diseases.

## Material and methods

Cell lines and reagents. HT-p21-9 cells are derivatives of HT1080 human fibrosarcoma cells, where p21 expression can be turned on or off using a physiologically neutral agent isopropyl--thio-galactosidase (IPTG) [16,49-51]. HT-p21-9 cells express GFP.  WI-38-Tert, WI-38 fibroblasts immortalized by telomerase were described previously [16,17]. Melanoma cell lines, MEL-9 (SK-Mel-103) and MEL-10 (SK-Mel-147), were described previously [52,53].  RPE cells were described previously [21,22]. MEF, mouse fibroblasts isolated from 13-day embryos, were provided by Marina Antoch (RPCI) and maintained in DMEM supplemented with 10% FCS. Rapamycin (LC Laboratories, MA, USA), IPTG (Sigma- Aldrich, St. Louis, MO), nutlin-3a (Sigma-Aldrich) were used as previously described [17].

Lentiviral shRNA construction . Bacterial glycerol stocks [clone NM_000548.2-1437s1c1 (#10), NM_000548.x-4581s1c1 (#7) and NM_000548.2-4551s1c1 (#9)] containing lentivirus plasmid vector pLKO.1-puro with shRNA specific for TSC2 was purchased from Sigma. The targeting sequences are: CCGGGCTCATCAACAGGCAGTTCTACTCGAGTAGAACTGCCTGTTGATGAGCTTTTTG (#10), CCGG CAATGAGTCACAGTCCTTTGACTCGAGTCAAAGGACTGTGACTCATTGTTTTTG (#7) and  CCGGCGACGAGTCAAACAAGCCAATCTCGAGATTGGCTTGTTTGACTCGTCGTTTTTG (#9).

pLKO.1-puro lentiviral vector without shRNA was used as a control. Lentiviruses were produced in HEK293T cells after co-transfection of lentivirus plasmid vector with shRNA or control vector with packaging plasmids using Lipofectamine2000 (Invitrogen). After 48h and 72h medium containing lentivirus was collected, centrifuged at 2000g and filtered through 0.22 uM filter. Filtered virus containing medium was used for cell infection or stored at -80 C. Cells were transduced with lentivirus in the presence of 8 mg/ml polybrene and selected with puromycin (1-2 mg/ml) for 4-6 days. Cells were treated with drugs either 24h after transduction or after puromycin selection for infected cells.

Colony formation assay. Plates were fixed and stained with 1.0 % crystal violet (Sigma-Aldrich).

Immunoblot analysis. The following antibodies were used: anti-p53 and anti-p21 antibodies from Cell signaling and anti-actin antibodies from Santa Cruz Biotechnology, rabbit anti-phospho-S6 (Ser240/244) and (Ser235/236), mouse anti-S6, mouse anti-phospho- p70 S6 kinase (Thr389), mouse anti-p21, rabbit anti-phospho-4E-BP1 (Thr37/46) from Cell Signaling; mouse anti-4E-BP1 from Invitrogen; mouse anti-p53 (Ab-6) from Calbiochem.

Beta-galactosidase staining. beta-Gal staining was performed using Senescence -galactosidase staining kit (Cell Signaling Technology) according to manufacturer's protocol.

## Supplementary figures

Supplementary Figure 1 Depletion of TSC2 converts quiescence into senescence in HT-p21-9 cells. (**A**) HT-p21-9 cells were transduced with control lentivirus (pLKO) or lentivirus expressing shTSC2 (sequence # 7, 8, 9) and selected with puromycin for 10 days and then immunoblot was performed. (**B**) HT-p21-9 cells were transduced with control pLKO or shTSC2  (and selected for 4 days with puromycin). Then 1000 cells were plated per 60-mm dishes and, the next day, were treated with nutlin-3a for 3 days. Then nutlin-3a was washed out and cells were cultivated in fresh medium for 8 days.  Colonies were stained with crystal violet.

Supplementary Figure 2 Irreversible and reversible effects of nutlin-3a and rapamycin:. Mel-10 and Mel-9 cells were incubated with 10 uM nutlin (N) and 500 nM rapamycin (R) for 4 day and then nutlin-3a was washed. After a week, cells were counted.
