# Supplementary figures and images for: The choice between p53-induced senescence and quiescence is determined in part by the mTOR pathway
Source: Aging (Albany NY). 2010 Jun 25;2(6):344–52. doi: 10.18632/aging.100160 (PMC2919254; doi:10.18632/aging.100160)

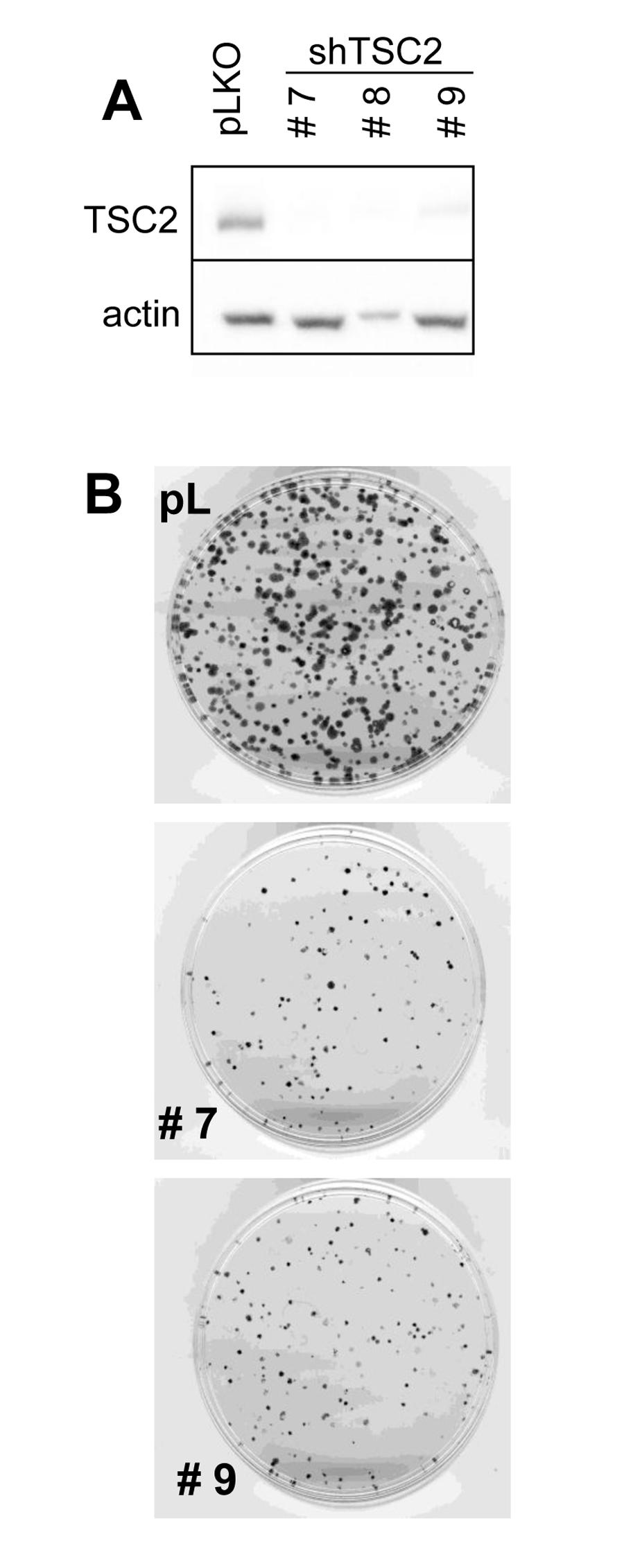

Supplement: Supplementary Figure 1 — (A) HT-p21-9 cells were transduced with control lentivirus (pLKO) or lentivirus expressing shTSC2 (sequence # 7, 8, 9) and selected with puromycin for 10 days and then immunoblot was performed. (B) HT-p21-9 cells were transduced with control pLKO or shTSC2 (and selected for 4 days with puromycin). Then 1000 cells were plated per 60-mm dishes and, the next day, were treated with nutlin-3a for 3 days. Then nutlin-3a was washed out and cells were cultivated in fresh medium for 8 days. Colonies were stained with crystal violet. [file aging-02-344-s001.tif]

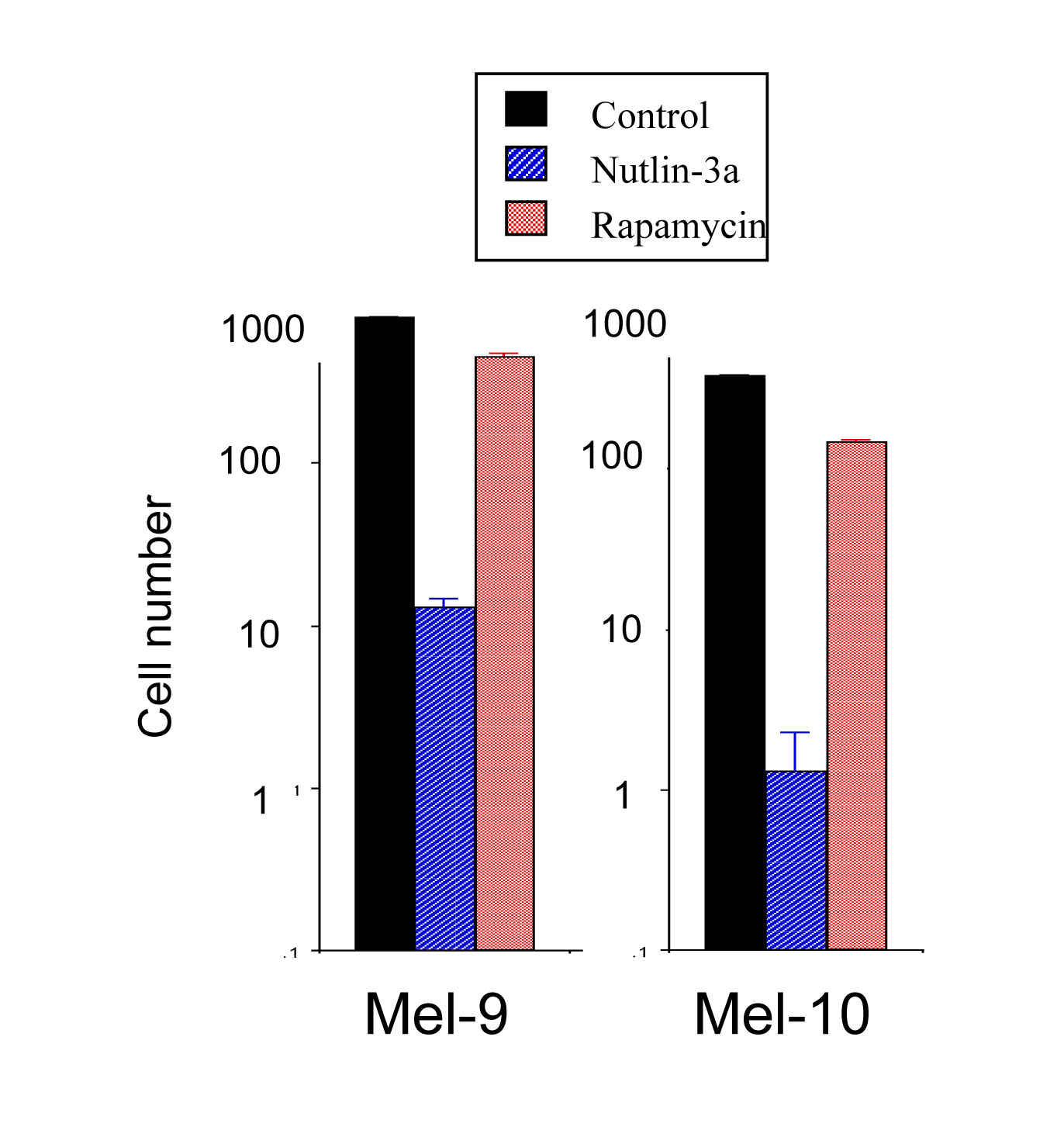

Supplement: Supplementary Figure 2 — Mel-10 and Mel-9 cells were incubated with 10 uM nutlin (N) and 500 nM rapamycin (R) for 4 day and then nutlin-3a was washed. After a week, cells were counted. [file aging-02-344-s002.tif]
